# Persistent cognitive slowing in post-COVID patients: longitudinal study over 6 months

**DOI:** 10.1007/s00415-023-12069-3

**Published:** 2023-11-07

**Authors:** Eva Maria Martin, Annie Srowig, Isabelle Utech, Simon Schrenk, Fabian Kattlun, Monique Radscheidt, Stefan Brodoehl, Peter Bublak, Matthias Schwab, Christian Geis, Bianca Besteher, Philipp A. Reuken, Andreas Stallmach, Kathrin Finke

**Affiliations:** 1 Department of Neurology, Jena University Hospital, Jena, Germany; 2 Department of Psychiatry, Jena University Hospital, Jena, Germany; 3 German Center for Mental Health (DZPG), Jena, Germany; 4 Department of Internal Medicine IV (Gastroenterology, Hepatology and Infectious Diseases), Jena University Hospital, Jena, Germany; 5 Center for Sepsis Control and Care, Jena University Hospital, Jena, Germany; 6 Department of Psychology, Ludwig Maximilian University of Munich, Munich, Germany

**Keywords:** Post-COVID, Tonic alertness, Processing speed, Fatigue, Cognitive dysfunction, Longitudinal course

## Abstract

**Background:**

Fatigue is a frequent and one of the most debilitating symptoms in post-COVID syndrome (PCS). Recently, we proposed that fatigue is caused by hypoactivity of the brain’s arousal network and reflected by a reduction of cognitive processing speed. However, it is unclear whether cognitive slowing is revealed by standard neuropsychological tests, represents a selective deficit, and how it develops over time.

**Objectives:**

This prospective study assesses whether PCS patients show deficits particularly in tests relying on processing speed and provides the first longitudinal assessment focusing on processing speed in PCS patients.

**Methods:**

Eighty-eight PCS patients with cognitive complaints and 50 matched healthy controls underwent neuropsychological assessment. Seventy-seven patients were subsequently assessed at 6-month follow-up. The Test for Attentional Performance measured tonic alertness as primary study outcome and additional attentional functions. The Neuropsychological Assessment Battery evaluated all key cognitive domains.

**Results:**

Patients showed cognitive slowing indicated by longer reaction times compared to control participants (*r* = 0.51, *p* < 0.001) in a simple-response tonic alertness task and in all more complex tasks requiring speeded performance. Reduced alertness correlated with higher fatigue (*r* =  − 0.408,* p* < 0.001). Alertness dysfunction remained unchanged at 6-month follow-up (*p* = 0.240) and the same was true for most attention tasks and cognitive domains.

**Conclusion:**

Hypoarousal is a core deficit in PCS which becomes evident as a selective decrease of processing speed observed in standard neuropsychological tests. This core deficit persists without any signs of amelioration over a 6-month period of time.

**Supplementary Information:**

The online version contains supplementary material available at 10.1007/s00415-023-12069-3.

## Introduction

It is now well established that post-COVID syndrome (PCS) represents a serious complication in a substantial number of patients following SARS-CoV-2 infection. PCS is diagnosed when COVID-19-related symptoms persist for more than 3 months [20]. It can occur even after an initially mild to moderate course of infection [1, 8], and comprise a large variety of symptoms. Around 30% of PCS patients show neurological and neuropsychiatric sequelae [23], such as fatigue, depressive symptoms, and cognitive dysfunction [2, 4, 22, 29]. These are experienced as particularly debilitating, as they have detrimental effects on daily functioning in PCS patients and hamper a successful return to their jobs [17].

Recently, we proposed that fatigue is caused by hypoactivity within the brain’s arousal network, reflected by a fundamental slowing of processing speed in PCS patients [12]. In particular, the variance of (visual) processing speed in a laboratory task was explained by a neurophysiological measure of central nervous arousal, i.e., pupillary unrest, and the level of subjective mental fatigue. Thus, based on our data, processing speed not only seems to represent a reliable measure of the brain’s activation level, but it can also provide an objective proof of the subjective feeling of mental fatigue.

Although these results may represent an important step towards a better understanding of the neurocognitive deficits in PCS patients, a number of questions arise from this preceding study: first, it remains unclear whether a reduction of processing speed can be evidenced in standard neuropsychological assessment procedures as well. Given the high prevalence of PCS, the suitability of conventional, clinically established tests for disclosing processing speed deficits would be of great practical significance. Second, in our preceding study [12], we were not able to assess whether cognitive domains other than processing speed are also affected by the underlying arousal deficit. A demonstration that processing speed is selectively impaired in PCS patients would bear strong support for our hypoarousal model. Finally, it remains unknown whether hypoarousal is a temporary and transient, or a persisting problem in PCS patients. This is an important question for estimating the probability whether PCS patients are able to re-uptake their job in the near future. However, while subjective complaints of fatigue are known to prevail even after 2 years [25], follow-up observations including an objective measure of fatigue are still lacking. Hence, longitudinal assessment of processing speed is urgently required.

Thus, the first aim of the present study was to analyze whether a reduction of processing speed in PCS patients is evidenced in conventional neuropsychological tests as applied in a standard clinical setting. We compared PCS patients with cognitive complaints to sociodemographically matched healthy control participants and used tonic alertness, measured in a clinically established simple-response task, as our primary outcome. The second aim was to assess whether reduced processing speed is a selective deficit in PCS patients. We employed a computerized reaction time-based neuropsychological assessment procedure and a comprehensive test battery to explore whether the overall cognitive profile across different relevant cognitive domains would be indicative of particular deficits in tasks reliant on fast information processing speed. As secondary outcomes, we explored whether speed-dependent measures in general, such as reaction times and test scores relying on task completion times, were compromised in PCS patients. Additionally, we investigated whether test scores less dependent on processing speed were less affected. Third, to corroborate the relationship between hypoarousal and fatigue, we investigated whether performance in the primary outcome tonic alertness simple-response measure and the other measures that tapped processing speed was related to the degree of experienced fatigue. As a fourth aim of our study, we included a follow-up assessment after 6 months to evaluate whether processing speed deficits persist or ameliorate over time. To our knowledge, this is the first longitudinal study that particularly addresses processing speed, i.e., alertness dysfunction in PCS patients with cognitive complaints.

## Methods

### General procedure

**Fig. 1:**
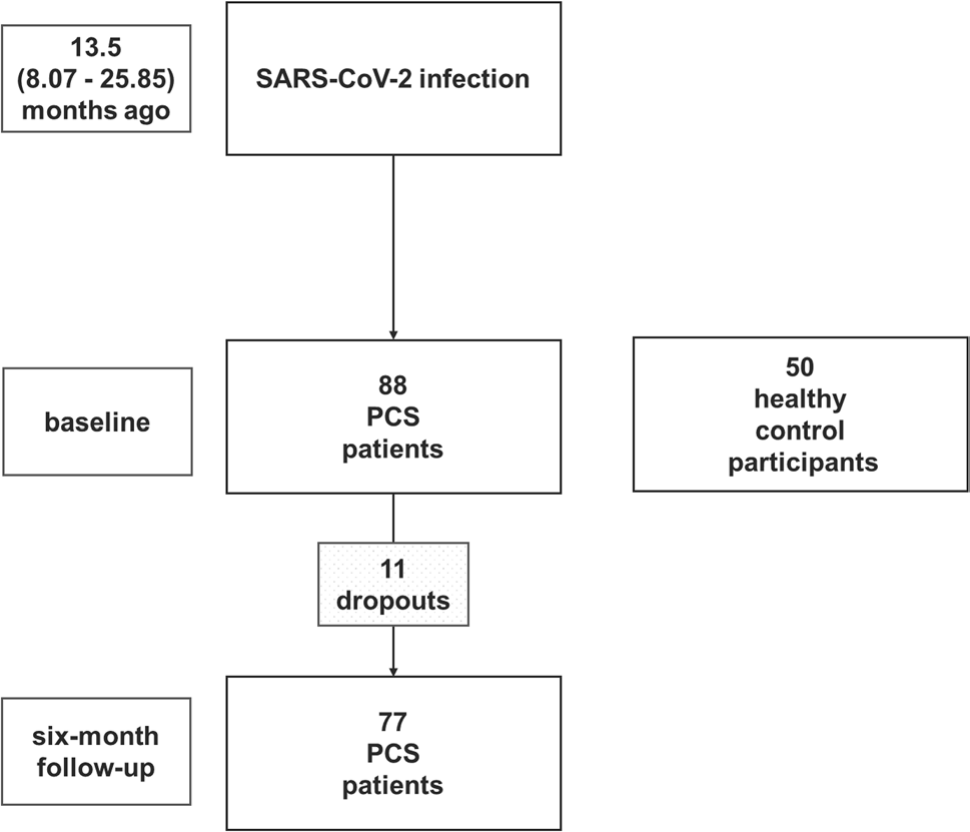
Study flow: 88 PCS patients and 50 sociodemographically matched healthy control participants were assessed with neuropsychological tests during the period from January 21, 2021 to September 27, 2022 at the Neuro-Post-COVID-Centre of the Department of Neurology of Jena University Hospital. Seventy-seven PCS patients returned for the 6-month follow-up assessment

Eighty-eight patients, fulfilling the NICE criteria for PCS [20], with subjective cognitive dysfunction after polymerase chain reaction confirmed SARS-CoV-2 infection, who presented for the first time from January 21, 2021 to September 27, 2022 at the Neuro-Post-COVID-Centre of the Department of Neurology of Jena University Hospital (JUH), were prospectively included in the study (Fig. 1). Patients underwent comprehensive clinical examination by a neurologist in order to confirm the PCS NICE criteria and to exclude alternative medical explanations for cognitive dysfunction before inclusion. To determine if the patient sample was adequately sized for longitudinal analysis, a sample size calculation using G*power 3.1.9.6 [7] was conducted. Expecting small effect sizes for the neurocognitive changes (*d* = 0.4) with an *α*-level of 0.05 and power of 90%, the estimated minimum sample size was *n* = 71. The control sample consisted of 50 healthy participants without known prior SARS-CoV-2 infection according to self-report, matched for age, gender and education. Healthy control participants were recruited via online notices, were financially compensated for participating in the study and gave written informed consent before inclusion in the study. Inclusion criteria for patients and healthy participants were an age between 18 and 65 years, normal or corrected-to-normal vision and normal premorbid intelligence (IQ > 85 as estimated by the German vocabulary test MWT-B [11]). Exclusion criteria were any history of psychiatric (e.g., addiction and substance abuse, schizophrenia, bipolar disorder, major depression, obsessive compulsive disorder, anxiety disorder), neurological (e.g., epilepsy, multiple sclerosis, stroke) diseases prior to COVID-19, and/or any severe neurological condition (e.g., stroke) as a direct consequence of COVID-19.

Baseline assessment with neuropsychological standard, age-normed tests was carried out by trained neuropsychologists. Of the initial sample, 77 patients returned for a follow-up assessment including parallel versions of the neuropsychological tasks after 6 months (6-month follow-up) (Fig. 1). The 11 dropouts (12.5%) were due to the following reasons: No access granted to the hospital because of failing pandemic access criteria (*n* = 1), inpatient rehabilitation stay (*n* = 4), move to another state (*n* = 1), acute viral infection (*n* = 2), a family emergency (*n* = 1) and unknown reasons (*n* = 2). Our study followed the Helsinki II ethics regulations and was approved by the ethics committee of JUH (No. 5082–02/17).

### Assessment of sociodemographic and basic clinical data

Height, weight (for calculating *body mass index*), *nicotine use*, *education level* in school years and *occupational status* were assessed via self-report. Occupational status categories were: occupation not requiring specialized education/apprenticeship = 1; occupation requiring apprenticeship or current apprenticeship = 2; academic profession or current enrollment at university = 3. Comorbidities were assessed via self-report and, for patients, cross-referenced with medical records. The *Charlson Comorbidity Index* (CCI, [5]) was calculated to assess the general *burden of comorbidities*. The CCI ranges from 0 to 37, with higher values indicating a greater burden of comorbidities. It is a weighted sum score of 19 potential comorbid conditions. (Weighted with 1: myocardial infarct, congestive heart failure, peripheral vascular disease, cerebrovascular disease, dementia, chronic pulmonary disease, connective tissue disease, ulcer disease, mild liver disease, and diabetes. Weighted with 2: hemiplegia, moderate to severe renal disease, diabetes with end organ damage, cancer, leukemia, and lymphoma. Weighted with 3: moderate to severe degree of liver disease. Weighted with 6: metastatic cancer and acquired immunodeficiency syndrome).

### Neuropsychological assessment

Neuropsychological assessment took place on two consecutive days. Questionnaires and computerized assessment with the Test of Attentional Performance (TAP) were administered on one day and the screening module of the Neuropsychological Assessment Battery (S-NAB) on another day. In between tests, patients and healthy control participants could take short breaks, if necessary.

#### Test of attentional performance (TAP) subtests

The following age-normed subtests of the TAP [30, 31]) battery (version 2.3.1) were applied: The primary outcome *tonic alertness* as the relevant proxy of endogenous arousal was assessed in a simple-response task. Participants were asked to respond to a small cross-presented at the screen center by pressing a key as fast as possible. Reaction times (RTs) were measured.

As secondary outcomes, the following subtests were assessed: Phasic *alertness*, i.e., the temporary increase in alertness to process expected stimuli, was assessed in a cued version of this simple-response task where the cross was preceded by a warning sound. *Divided attention*, i.e., the ability to divide attentional capacity on simultaneously ongoing processes, was assessed in a test where participants had to monitor and to react to both stimuli in the visual and the auditory modality. Small crosses with changing locations where presented on the screen and participants had to press the key as fast as possible when four out of these crosses formed a square. In addition, a sequence of alternating high and low tones was presented and participants had to also respond with a key press when the same tone was presented twice. RTs and errors were measured. *Inhibition*, i.e., the ability to inhibit inappropriate responses, was measured in a go/nogo task. Participants were presented with two visual stimuli and were asked to only react to one of them by pressing a key as fast as possible. RTs and errors were measured. RTs hereby served as speed-dependent secondary outcome measures, while accuracy values served as outcomes not reflecting processing speed. All TAP measures (mean RTs and error scores) were converted into age-normed T-scores with *M* = 50 and *SD* = 10 provided by the test program. Total TAP assessment took approximately 25 min.

#### Screening module of the Neuropsychological Assessment Battery Screening (S-NAB)

Further secondary outcomes were the five subscores addressing different cognitive domains assessed with the 14 age-normed subtests of the German version of S-NAB [18]. Performance in some of these subscores relies more critically on fast performance, and thus, cognitive speed, than in others. Due to their stimulus material, time-on task limitations and their scoring rules, cognitive processing speed is relevant for performance in the domains of *attention* (measured via digit span forwards and backwards, time needed to perform visual search for letters A—discriminating letter A in a digit/letter array, digits, time needed to perform visual search for letters B—discriminating letter A in a digit/letter array while summing up digits) and *executive functions* (time needed to solve visual mazes, words produced in a 2 min verbal phonemic fluency task). Processing speed is less relevant in the domains of *language* (picture naming, scene description), *perception* (visual discrimination of abstract visual figures, design construction) and *memory* (visual learning—immediate and delayed recognition of shapes, verbal learning—immediate and delayed recall of a two-sentence story). Standard scores (*M* = 100, *SD* = 15) were calculated according to age norms provided in the test manual for all domains. Baseline assessment was conducted using parallel form one, the 6-month follow-up assessment was conducted using parallel form two. S-NAB assessment took approximately 45 min.

#### Questionnaires assessing fatigue and depressive symptoms

*Fatigue* was assessed by the Fatigue Assessment Scale (FAS) [13] which consists of 10 items on a 5-point-scale (1 = never, 5 = always). The total score ranges from 10 to 50, with higher scores indicating higher levels of experienced fatigue. *Depressive symptoms* were assessed using the depression subscale of the German version of the Hospital Anxiety and Depression Scale (HADS-D) [10, 21] which consists of seven items. Scores range between 0 and 24 with higher scores indicating higher levels of experienced depression.

### Statistical analysis

The standardized test norm scores obtained by PCS patients were first descriptively analyzed in order to reveal those tests where PCS patients, on average, performed below what was expected for their age based on the test norm samples. Then, for comparisons between PCS patients and healthy control participants, independent sample t-tests, Wilcoxon rank-sum tests, or *χ*2-tests were run as appropriate. Variables were previously inspected for normal distribution using Shapiro–Wilk tests. To assess the relationship between alertness and fatigue, and more generally the relationships between cognitive, sociodemographic and clinical measures within the PCS patient group at baseline, Pearson’s or point-biserial correlational analyses were conducted. For comparisons between baseline and 6-month follow-up assessment within the PCS patient group, two-tailed paired-samples t-tests or Wilcoxon signed rank tests (alpha = 0.05) were applied as appropriate. Missing values at 6-month follow-up were imputed using predictive mean matching. Results based on non-imputed data can be found in Supplement’s Sect. 4. Cohen's *d* values are reported for t-tests and *r* for Wilcoxon rank-sum and signed rank tests, indicating small (*d* ≥ 0.2; *r* ≥ 0.1), moderate (*d* ≥ 0.5; *r* ≥ 0.3) or large (*d* ≥ 0.8; *r* ≥ 0.5) effects. In addition, the percentage of individuals whose performance fell more than 1.5 *SD*s below the standard mean of the age norm sample of the respective test was calculated for the control group and the PCS patient group at baseline and at 6-month follow-up. We controlled for a false discovery rate (FDR) of 5% among all tests using the Benjamini–Hochberg method [27]. Statistical analyses were run using SPSS version 28.0.0.0 (190). Figures were created using R version 4.2.1.

## Results

### Sample description

Basic sociodemographic and clinical information of PCS patient data are presented in Table 1. At baseline assessment, on average 13.5 (8.07–25.85) months had passed since acute SARS-CoV-2 infection and 18.2% of patients had been treated at the hospital during acute COVID-19 disease. There were no significant differences between groups in terms of age (*t*(136) = 0.349, *p* = 0.728), education (*W* = 5821.00, *Z* =  − 1.52,* p* = 0.130), occupational status (*χ*^2^(2.00) = 2.300, *p* = 0.317), burden of comorbidities (CCI) (*W* = 5826.00, *Z* =  − 0.597,* p* = 0.550), nicotine use (*χ*^2^(1.00) = 1.486, *p* = 0.223), body mass index (*W* = 3024.50, *Z* =  − 1.10,* p* = 0.270), and sex ratios (*χ*^2^(1.00) = 0.601, *p* = 0.675). PCS patients showed higher fatigue ratings in the FAS (*W* = 6530.00, *Z* = 9.102,* p* < 0.001, *r* = 0.78) and higher depression ratings in the HADS-D (*W* = 3576.00, *Z* = 6.122,* p* =  < 0.001, *r* = 0.55) compared to healthy controls with large effect sizes.

**Table 1:** Sociodemographic and clinical data for PCS patients at baseline and healthy control participants

	PCS patients at baseline (*n* = 88)	Healthy control participants (*n* = 50)
Age (in years)		
*M (SD),* range: Missings:	46.67 (10.74), 21.00–64.00 0.00%	45.98 (11.95), 24.00–65.00 0.00%
Sex distribution		
Female:	76.1%	80.00%
Male:	23.9%	20.00%
Missings:	0.00%	0.00%
Education (in years)		
Mdn (IQR), range:	10.70 (2.00), 10.00–13.00	11.00 (2.00), 9.00–12.00
Missings:	0.00%	0.00%
Occupational status		
Academic profession:	27.30%	30.00%
Apprenticeship profession:	72.70%	62.00%
Untrained profession:	0.00%	2.00%
Missings:	0.00%	6.00%
Burden of comorbidities (CCI)		
Mdn (IQR), range	0.63 (0.89), 0.00–4.00	0.72 (0.98), 0.00–4.00
Missings:	0.00%	6.00%
Body mass index		
Mdn (IQR), range	26.71 (4.77), 18.50–37.34	25.92 (5.28), 17.92–42.24
Missings:	0.00%	0.00%
Nicotine use		
Regular use:	14.80%	22.00%
No use:	84.10%	72.0 0%
Missings:	1.10%	6.00%
Time from SARS-CoV-2 infection (in months)		
*M (SD*), range:	13.5 (4.6), 8.07–25.85	–
Missings:	0.00%	
Hospitalization		
Intensive care unit:	6.8%	–
Normal ward:	11.4%	
No hospitalization:	81.8%	
Missings:	0.00%	
Fatigue		
Mdn (IQR), range:	36.00 (11.00), 17.00–49.00	14.50 (6.00), 10.00–32.00
Missings:	0.00%	0.00%
Depressive symptoms		
Mdn (IQR), range:	7.00 (4.00), 0.00–15.00	2.00 (5.00), 0.00–10.00
Missings:	0.00%	0.00%

*M* Means, *SD* standard deviations, *Mdn* medians, *IQR* interquartile ranges or percentages for the PCS patient group at baseline and the healthy control participant group are reported as appropriate. Burden of comorbidities is Charlson Comorbidity Index (CCI), Fatigue is Fatigue Assessment Scale (FAS) score; Depressive symptoms is Hospital Anxiety and Depression Scale (HADS-D) depression subscore

### PCS patients’ performance in the primary outcome tonic alertness and secondary outcome measures relying on processing speed

**Fig. 2:**
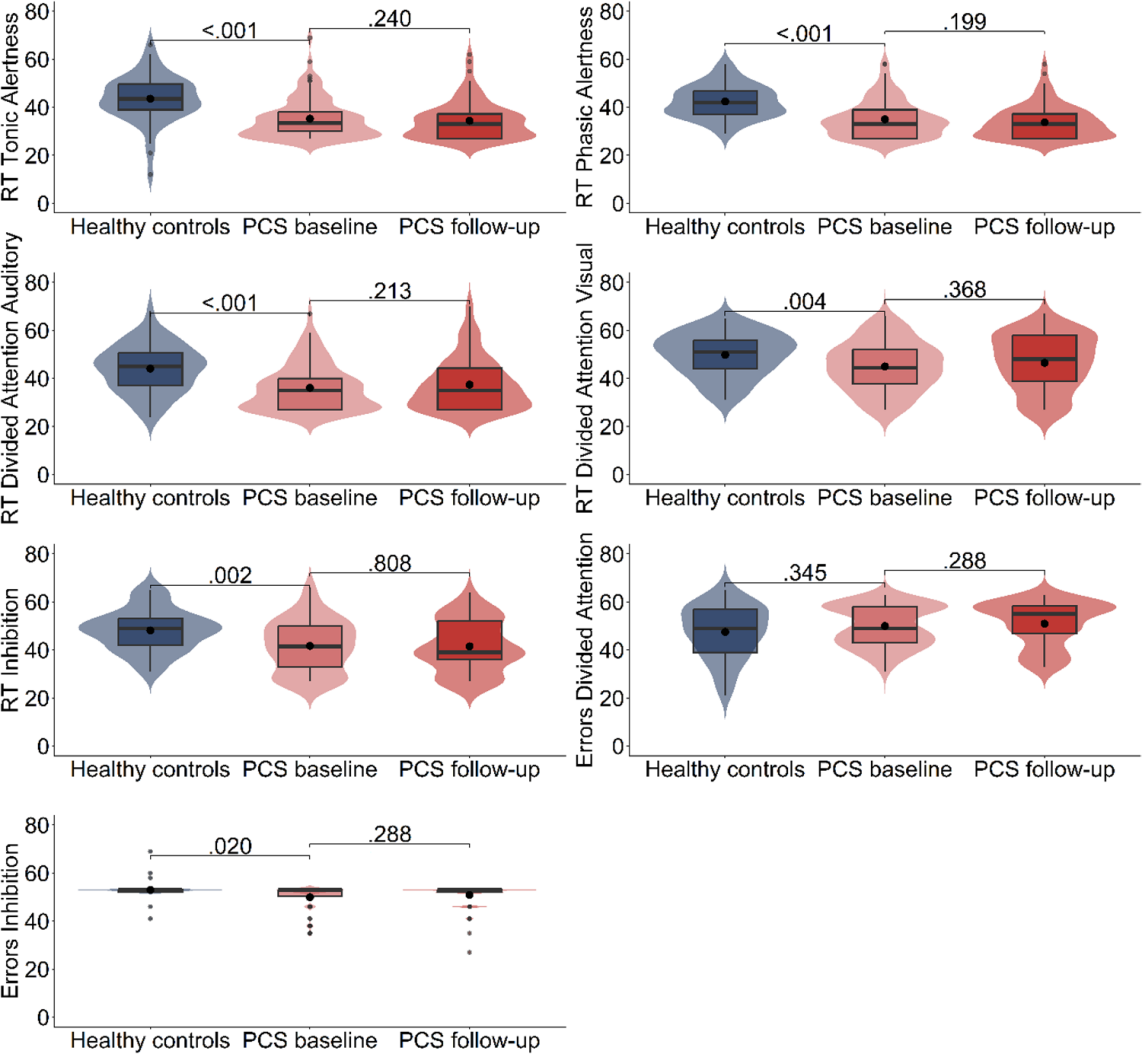
Violin plots of distributions, medians (line) and means (point) of T-scores of performance in the subtests of the Test for Attentional Performance (TAP) for healthy control participants (blue) and PCS patients at baseline (light red) and at 6-month follow-up (dark red). T-scores: *M* = 50, SD = 10. *P* values of comparisons between PCS patients and healthy control participants and between baseline and 6-month follow-up assessment within the PCS patient group

**Fig. 3:**
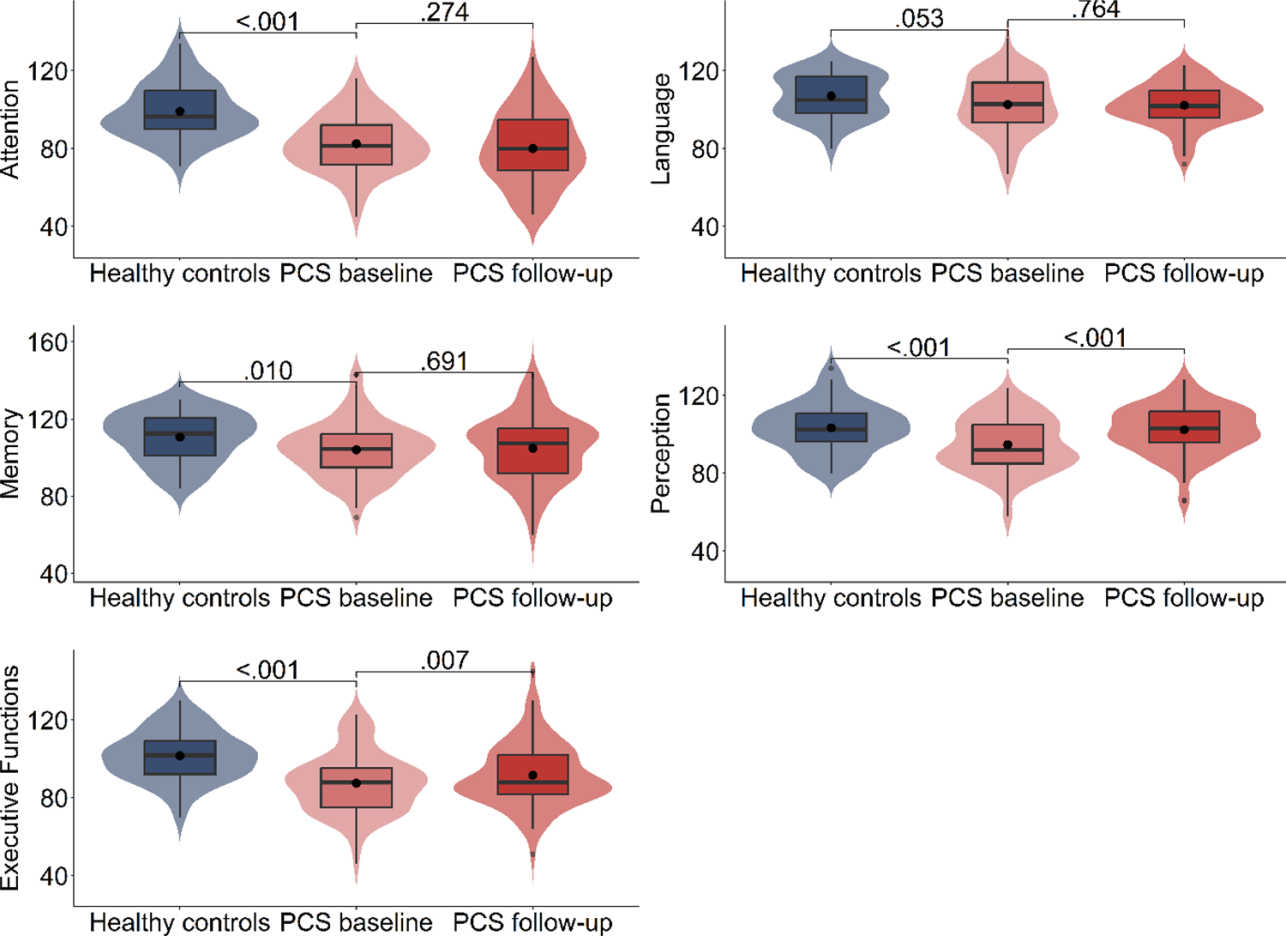
Violin plots of distributions, medians (line) and means (point) of standard scores of performance in the Neuropsychological Assessment Battery Screening (S-NAB) for healthy control participants (blue) and PCS patients at baseline (light red) and at 6-month follow-up (dark red). Standard scores: *M* = 100, *SD* = 15. *P* values of comparisons between PCS patients and healthy control participants and between baseline and 6-month follow-up assessment within the PCS patient group

The blue and light red violin plots presented in Figs. 2 and 3 illustrate mean performance and distribution of healthy control participants’ and PCS patients’ performance at baseline in the different neuropsychological tasks and cognitive domains. Table 2 lists the statistical values for comparisons between groups. Furthermore, the percentage of individuals per group whose performance fell more than 1.5 *SD*s below the standard mean of the respective age norm sample of the test is given.

**Table 2:** Comparison of age-normed scores of PCS patients and healthy control participants in the different test battery tasks and domains at baseline

Cognitive measures	PCS patients (*n* = 88)	Healthy control participants (*n* = 50)	Comparison between groups	
TAP—T-scores (*M* = 50, *SD* = 10)	Mdn (IQR) Percentage below > 1.5 *SD* from the norm’s mean	Mdn (IQR) Percentage below > 1.5 *SD* from the norm’s mean	*p*	*r*
RT tonic alertness	34.00 (8.00) 53.41%	43.50 (11.00) 12.00%	< 0.001	0.51
RT phasic alertness	33.00 (12.00) 59.09%	42.00 (10.00) 8.00%	< 0.001	0.50
RT divided attention (auditory)	35.00 (13.00) 48.86%	45.60 (15.00) 14.00%	< 0.001	0.38
RT divided attention (visual)	44.00 (15.00) 18.18%	51.50 (12.00) 8.00%	0.004	0.25
RT inhibition	41.50 (17.00) 27.27%	49.00 (12.00) 8.00%	0.002	0.29
Errors divided attention	49.00 (15.00) 1.14%	49.00 (18.00) 14.00%	0.345	
Errors inhibition	53.00 (7.00) 0.00%	53.00 (1.00) 0.00%	0.020	0.20
S-NAB—standard scores (*M* = 100, *SD* = 15)	*M* (*SD*) Percentage below > 1.5 *SD* from the norm’s mean	*M* (*SD*) Percentage below > 1.5 *SD* from the norm’s mean	*p*	*d*
Attention	82.52 (14.41) 36.6%	99.16 (14.10) 2.00%	< 0.001	1.17
Language	101.65 (17.34) 6.82%	107.06 (11.31) 0.00%	0.053	
Memory	104.09 (14.34) 2.27%	110.66 (12.63) 0.00%	0.010	0.48
Perception	94.72 (13.77) 9.09%	103.32 (12.57) 0.00%	< 0.001	0.65
Executive functions	87.61 (15.50) 28.41%	101.70 (12.79) 4.00%	< 0.001	0.97

*Mdn* and *IQR* are reported for Wilcoxon rank-sum tests, *M* and *SD* for *t* tests. FDR-adjusted *p* values. Effect sizes (Cohen’s *d* for *t* tests, *r* for Wilcoxon rank-sum tests) were only reported for significant tests with *d* ≥ 0.2; *r* ≥ 0.1 indicating small, *d* ≥ 0.5; *r* ≥ 0.3 indicating moderate, and *d* ≥ 0.8; *r* ≥ 0.5 indicating large effects

*TAP* Test for attentional performance, *S-NAB* Neuropsychological assessment battery screening module

First, the mean tonic alertness T-value, as the primary outcome measure indicative of hypoarousal, indicates that, on average, PCS patients responded around 1.5 *SD*s slower than expected for their respective age group according to the test norms. As listed in Table 2, more than half of the PCS patients scored 1.5 *SD*s below the test norms’ mean (note that a majority of 78.41% scored more than 1 *SD* below the norm’s mean, not listed in the table). The two groups’ interquartile ranges of RTs in the tonic alertness task have no overlap and values are clustered around the median of the respective group (PCS patients: Mdn = 34.00, IQR = 8.00; healthy control participants: Mdn = 43.50, IQR = 11.00), indicating a relatively consistent performance within each group (Fig. 2). The statistical comparison between the groups further validates this observation (*W* = 4892.500, *Z* = 0 − 5.934), as a large effect size was found for tonic alertness (see Table 2 for *p* and *r* values).

Around half of the PCS patients also showed particular slow results in the phasic alertness task and in the auditory condition of the divided attention task. The RT distributions depicted in Fig. 2 imply slower RTs of PCS patients than control participants in all other TAP battery subtasks. In the phasic alertness task, there is only minimal overlap of interquartile ranges and most values are clustered around the median of the respective group, also suggesting relatively homogeneous performance within groups. This is also confirmed by the statistical comparisons: A large effect size was found for the phasic alertness task (*W* = 4848.00, *Z* =  − 5.841); less pronounced differences were found for more complex attention tasks, with small to moderate effects for divided attention (auditory condition: *W* = 5112.00, *Z* =  − 4.473, visual condition: *W* = 5453.500, *Z* =  − 2.937) and inhibition (*W* = 5357.500, *Z* =  − 3.366) (see Table 2).

Two domain scores, namely the attention and executive function domain scores, heavily rely on speeded performance, and thus reflect processing speed. The mean standard scores imply that PCS patients perform, on average, more than one *SD* below their respective age norm group in the domain of attention and close to one *SD* below the norm in the domain of executive functions. In attention, more than a third, and in executive functions, a bit less than a third of the PCS patients scored 1.5 *SD*s below the test norms’ mean. The violin plots in Fig. 3 show that the task performance distributions of PCS patients and healthy control participants in the subdomains of attention and executive functions clearly differed. Similarly to the attention tasks of the TAP, the interquartile ranges of both groups had only minimal overlap, with values being centered around the respective group’s median. This was confirmed by the statistical comparisons (attention: *t*(136) =  − 6.576; executive functions: *t*(136) =  − 5.452) that revealed large effect sizes in both domains (see Table 2).

### PCS patients’ performance in secondary outcome measures not critically depending on processing speed

In contrast to the deficient reaction times, PCS patients’ TAP task accuracy distributions were quite comparable to those of the control participants as depicted in the violin plots of Fig. 2. Accordingly, no difference was found for the error rates in the divided attention task (*W* = 3239.500, *Z* =  − 0.943); and only a slightly enhanced error rate with a small effect size was found in the inhibition task (*W* = 5648.000, *Z* =  − 2.390) (see Table 2).

In the S-NAB, the subdomain scores of perception, memory and language rely on accuracy rather than speed of performance. Performance in the PCS group was lower compared to the control group in perception (*t*(136) = -3.640) and in memory (*t*(136) =  − 2.698). However, in these domains, the effect sizes implied less pronounced group differences, with a moderate effect for perception, and a small effect for memory. No group difference was found concerning the domain of language* t*(136) =  − 1.977) (see Table 2).

Notably, the reported results were also found when the 16 hospitalized PCS patients were excluded from the analyses (see Supplement’s Sect. 1, Table 1) and when regression models were employed with group as a factor and baseline characteristics as predictors for test performance (see Supplement’s Sect. 2, Table 2).

### Associations of tonic alertness and other outcomes tapping on processing speed with the degree of fatigue and with potentially relevant sociodemographic and clinical variables

**Fig. 4:**
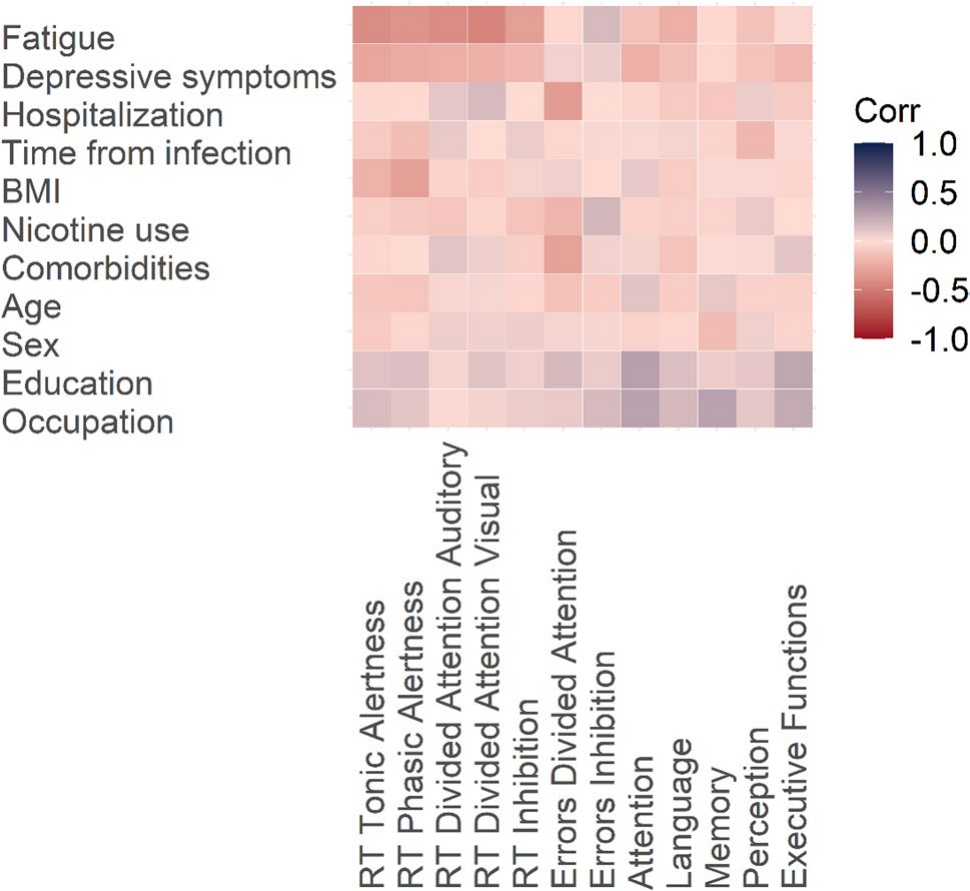
Heat map depicting the strength of Pearson/point-biserial correlations between neurocognitive performance (with higher values in standardized scores relating to better performance) and self-rated fatigue, self-rated depressive symptoms, hospitalization, time from SARS-CoV-2 infection, body mass index, nicotine use, comorbidities, sex, age, education, and occupational status within the PCS patient group at baseline assessment. *RT* reaction times

The third aim of this study was to investigate whether performance in the tonic alertness simple-response measure and other speed-dependent outcome measures was related to the degree of experienced fatigue. In the heat map provided in Fig. 4, the strength of the relations between the different neuropsychological scores obtained from PCS patients and their fatigue ratings, as well as other relevant clinical and sociodemographic variables, are depicted in a color-coded manner (see Table 3 in Supplement’s section 3.1) for all respective correlation coefficients). The left upper line of the heat map shows the relationship between fatigue and RTs in the tonic alertness and other TAP tasks.

Concerning the primary outcome, lower performance, i.e., longer RTs, in the tonic alertness task were indeed correlated with higher fatigue ratings (*r* =  − 0.408, *p* < 0.001). That is, the subjectively experienced and the measurable neurocognitive proxies of hypoarousal were related to each other.

Similarly, other speed-dependent secondary outcome measures in the TAP were associated with fatigue: Higher fatigue ratings were correlated with longer RTs in the phasic alertness task (*r* =  − 0.376, *p* = 0.020), the divided attention task in the auditory (*r* =  − 0.414, *p* < 0.001) and the visual stimulus condition (*r* =  − 0.459, *p* < 0.001).

No relationship was found between potentially relevant sociodemographic and clinical variables, i.e., depressive symptoms, hospitalization, time from SARS-CoV-2 infection, BMI, nicotine use, burden of comorbidities, age, sex, education and occupational status with neuropsychological measures at baseline.

### Longitudinal course of processing speed deficits in PCS patients: comparison of baseline vs. 6-month follow-up

The red violin plots presented in Figs. 2 and 3 also illustrate mean performance and distribution of PCS patients’ performance in the different neuropsychological tasks at baseline (light red) and 6-month follow-up (dark red). Table 3 lists the statistical values of the comparisons between the two time points.

**Table 3:** Comparison of PCS patients’ neurocognitive performance at baseline and at 6-month follow-up

Cognitive measures	Baseline	6-month follow-up	Comparison	
TAP—T-scores (*M* = 50, *SD* = 10)	Mdn (IQR) Percentage below > 1.5 *SD* from the norm’s mean	Mdn (IQR) Percentage below > 1.5 *SD* from the norm’s mean	*p*	*r*
RT tonic alertness	34.00 (8.00) 53.41%	33.00 (11.00) 54.55%	0.250	
RT phasic alertness	33.00 (12.00) 59.09%	33.00 (11.00) 63.63%	0.230	
RT divided attention (auditory)	35.00 (13.00) 48.86%	35.00 (18.00) 46.59%	0.277	
RT divided attention (visual)	44.00 (15.00) 18.18%	48.00 (20.00) 17.05%	0.231	
RT inhibition	41.50 (17.00) 27.27%	39.00 (16.00) 23.86%	0.808	
Errors divided attention	49.00 (15.00) 1.14%	55.00 (15.00) 6.82%	0.277	
Errors inhibition	53.00 (7.00) 0.00%	53.00 (1.00) 1.14%	0.277	
S-NAB—standard scores (*M* = 100, *SD* = 15)	*M* (*SD*) Percentage below > 1.5 *SD* from the norm’s mean	*M* (*SD*) Percentage below > 1.5 *SD* from the norm’s mean	*p*	*d*
Attention	82.52 (14.41) 36.6%	80.12 (18.78) 45.46%	0.277	
Language	101.65 (17.34) 6.82%	102.34 (10.88) 2.27%	0.798	
Memory	104.09 (14.34) 2.27%	104.91 (16.77) 6.82%	0.708	
Perception	94.72 (13.77) 9.09%	102.32 (12.46) 3.41%	< 0.001	0.47
Executive functions	87.61 (15.50) 28.41%	91.66 (16.20) 14.77%	0.013	0.34

*Mdn* and *IQR* are reported for Wilcoxon rank-sum tests, *M* and *SD* for *t* tests. FDR-adjusted *p* values. Effect sizes (Cohen’s *d* for *t* tests, *r* for Wilcoxon rank-sum tests) were only reported for significant tests with *d* ≥ 0.2 < 0.5 indicating small large effects

*TAP* Test battery for attentional performance, *S-NAB* Neuropsychological assessment battery screening module

Concerning the primary outcome, Fig. 2 shows that the distribution of the deficient performance in the tonic alertness task was highly similar at both time points. Accordingly, no statistical improvement (*Z* =  − 1663) was found (Table 3). The same holds true for the remaining speed-dependent measures of the TAP battery, i.e., RTs in the phasic alertness (*Z* =  − 1934), divided attention (auditory: *Z* =  − 1.304; visual: *Z* =  − 1.807) and inhibition tasks (*Z* = -0.243): The plots show that the performance level and distribution were comparable at both time points, and none of the comparisons revealed changes (see Table 3).

With respect to speed-dependent measures of the S-NAB, Fig. 3 shows that performance level and distribution changed slightly in the domain of executive functions while it did not change in the domain of attention (*t*(87) = 1.537). Accordingly, as listed in Table 3, an improvement was found in executive functions (*t*(87) =  − 3.209), albeit with a small effect size (see Table 3).

With respect to the measures that do not rely on processing speed, i.e., the TAP accuracy data (errors divided attention: *Z* =  − 1.337; errors inhibition: *Z* =  − 1.306) and the remaining S-NAB scores (language: *t*(87) =  − 0.369; memory:* t*(87) =  − 0.528), only for the S-NAB domain of perception (*t*(87) =  − 4.417) a slight improvement compared to baseline with a small effect size was found (see Table 3). Furthermore, no changes were found when comparing self-ratings for fatigue at the two time points (fatigue at baseline: Mdn = 36.00, IQR = 11.00; fatigue at follow-up: Mdn = 35.00, IQR = 16.00; *Z* =  − 0.643, *p* = 0.676).

### Correlation between individual processing speed performance at baseline and at 6-month follow-up

**Fig. 5:**
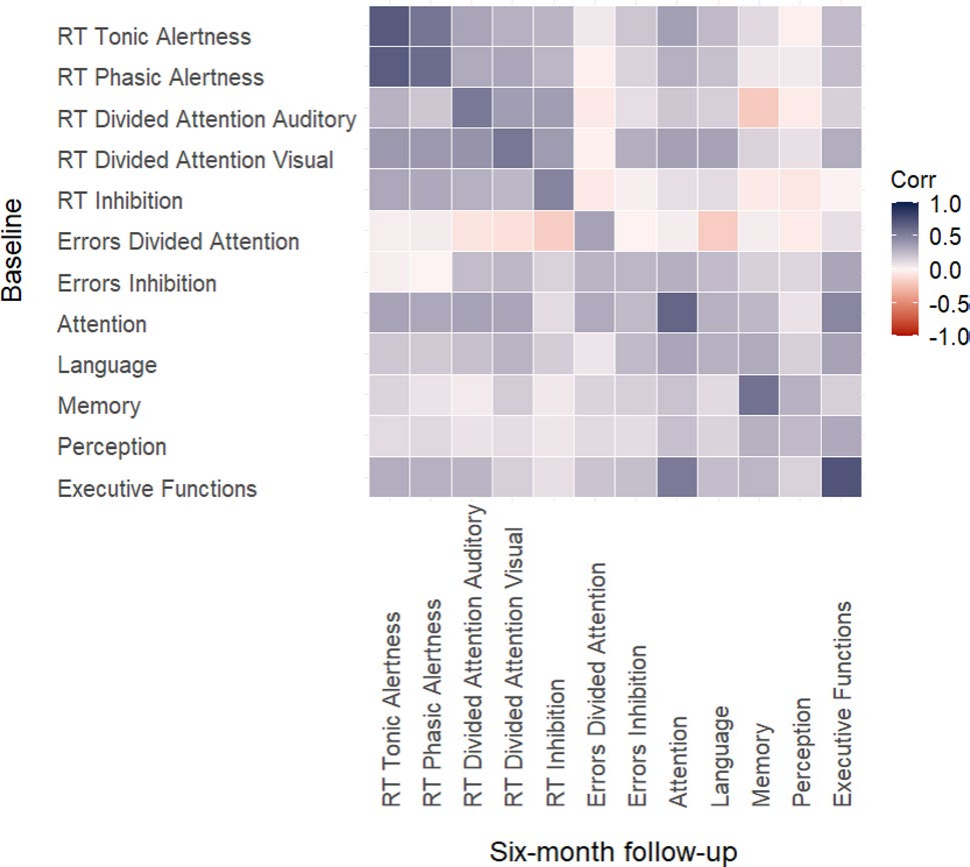
Heat map depicting the strength of Pearson correlations between neurocognitive performance at baseline and at 6-month follow-up within the PCS patient group. *RT* reaction times

In Fig. 5, the diagonal of the heat map illustrates the strengths of the relations between neurocognitive performance in the different tasks and domains of PCS patients at baseline and 6-month follow-up in a color-coded manner. These can be taken as a measure of the stability of the individual processing speed deficit.

Concerning the primary outcome, the upper left square shows that particularly the correlation between RTs in the tonic alertness task was high (*r* = 0.691, *p* < 0.00). The same was true for the secondary speed-dependent outcomes in the TAP: RTs in the phasic alertness task (*r* = 0.604, *p* < 0.001) and in divided attention tasks (auditory, *r* = 0.543, *p* < 0.001; visual, *r* = 0.552, *p* < 0.001) were highly, and RTs in the inhibition task (*r* = 0.487, *p* < 0.001) were moderately correlated. Also in the S-NAB, strong intercorrelations were found for those measures that rely on fast processing speed, i.e., in the subtasks of attention (*r* = 0.640, *p* < 0.001) and executive functions (*r* = 0.723, *p* < 0.001).

For the measures that are less dependent on fast processing, less pronounced intercorrelations implied less stability. We found weak-to-moderate correlations for the TAP tasks’ error rates (divided attention, *r* = 0.354, *p* = 0.001; inhibition, *r* = 0.264, *p* = 0.014). With respect to the S-NAB, weak correlations were found for language (*r* = 0.291, *p* = 0.007) and perception (*r* = 0.245, *p* < 0.001). The correlation for memory was the only exception among these tasks, as it was high between baseline and 6-month-follow-up (*r* = 0.572, *p* = 0.021). Coefficients of all correlations can be found in Table 4 in Supplement’s section 3.2.

## Discussion

According to the present study’s results, the cognitive performance profile of PCS patients with cognitive complaints using established, normed clinical neuropsychological tests aligns with the previous assumption of an underlying hypoarousal state [12]. This substantiates the tests’ utility in confirming and understanding cognitive impairment due to hypoarousal and accurate diagnosis. Furthermore, the longitudinal analyses revealed that neurocognitive deficits, especially tonic alertness dysfunction, persist over a time period of 6 months.

First, a reduction in processing speed was observed in the PCS patients in standard neuropsychological assessment procedures. Namely, particularly poor performance was found, with below-norm average values of more than half of the PCS patients in the simple-response task of the TAP battery reflecting reduced tonic alertness. In addition, the comparison to the healthy control group revealed a large effect size, indicating substantially lower tonic alertness in PCS patients compared to sociodemographically matched healthy control participants. Therefore, a computerized simple-response measure seems to be a highly appropriate neuropsychological tool for demonstrating reduced processing speed in PCS patients.

Furthermore, under-average performance compared to test norms and/or moderate to high effect sizes in comparison to the control group were generally found across measures relying on speeded performance and response time measurement. These measures included the phasic alertness task and the divided attention task of the TAP battery as well as the attention and the executive functions subscore of the S-NAB battery. Arguably, in these tasks with higher stimulus and instruction complexity, general cognitive slowing leads to delayed execution of each relevant subprocess in information processing and the response execution hierarchy, and finally to extended task execution times. Therefore, underperformance in these tasks can be attributed to low processing speed as the underlying deficient mechanism.

Second, domain subscores and task accuracy measures relying less on speeded performance were not or less affected, implying that processing speed is selectively impaired in PCS patients: Within the S-NAB battery, the language score was comparable to the healthy participants. The memory score was lower than in the control group, but the effect size was small and the average score of the patients indicated normal performance in reference to the test norms. The latter holds also true for the accuracy values (error rates) in the different attention subtasks of the computerized TAP battery. This specific profile of impaired speeded and unimpaired non-speeded tasks supports the assumption that the major contributor to neuropsychologically objectifiable cognitive deficits is, indeed, cognitive slowing due to hypoarousal of the brain.

Third, a clear relationship was found between the severity of fatigue and of cognitive slowing: higher fatigue (as an indicator of subjectively experienced hypoarousal [12]) was related to lower tonic alertness scores in PCS patients. Notably, higher fatigue ratings were also associated with higher reaction times in further subtasks in the computerized TAP battery. This relationship between high levels of experienced fatigue and slowed performance was specific, as it was not found for any sociodemographic variables (i.e., sex, age, education, and occupational level) or clinical values (i.e., depressive symptoms, time from SARS-CoV-2 infection, hospitalization, BMI, nicotine use, burden of comorbidities). This specific relationship affirms that the simple-response tasks validly reflect the major subjective complaint of PCS patients with neurological sequelae.

To sum up, our baseline assessment findings give support to the assumption of general cognitive slowing, i.e., reduced tonic alertness due to hypoarousal, as a core and selective cognitive deficit in neurological PCS patients. From a methodological point of view, our results indicate that simple-response time measurement represents an appropriate tool to objectify fatigue as a core clinical complaint in PCS patients. This has also been reported for other neurological disorders accompanied by fatigue such as multiple sclerosis [26].

Fourth, with respect to the critical question concerning the course of processing speed deficits in PCS patients, the longitudinal analyses revealed a persisting and relatively stable deficit profile implying a stable and selective deficit of processing speed across a 6-month period of time. Crucially, we did not find any indication of amelioration of tonic alertness dysfunction in the within-participant comparison or with respect to the percentage of participants showing particular poor performance. The intercorrelation specifically between alertness performances at the two time points was high. Thus, patients with more severe initial hypoarousal are also those who present with more pronounced impairment half a year later on. The only evidence for a slight improvement of performance across the two comprehensive neurocognitive batteries was found for the domains of perception and executive functions. As the performance of PCS patients in the domain of perception lay, on average, within the normal range at baseline, this improvement does not necessarily reflect actual amelioration. The executive functions score, indicating slowed planning and problem solving, might be of more relevance. While around a third of the PCS patients showed such deficient performance, this number halved by 6-month follow-up.

In previously published research, some cross-sectional results in participants following COVID-19 implied improvement of attentional performance with increasing time from SARS-CoV-2 infection. However, these participants were not necessarily complaining of long-lasting cognitive deficits [28]. Other cross-sectional studies did not report such relationship (e.g., [3, 9, 12]). The longitudinal findings of the present study indicate that, overall, there was no significant change in tonic attentional alertness, and, thus, in cognitive processing speed. The longitudinal results in our sample substantially go beyond the prior cross-sectional evidence for lack of spontaneous remission in PCS patients. Our results add critical within-patient evidence indicating chronification of cognitive deficits, especially of tonic alertness dysfunction, in PCS patients.

Reduced cognitive processing speed is known to have a severe negative impact on basic daily and occupational activities such as driving or reading (e.g., [14, 15, 19, 24]). Thus, our findings lead to the prognosis that the ability to return to their workspace might be severely limited in the long-term in a substantial part of PCS patients suffering from cognitive dysfunction. These results highlight the pressing demand for effective treatment specifically targeting hypoarousal in PCS patients. Of note, a computerized cognitive alertness training intervention was found to be effective in enhancing processing speed in elderly participants [16]. Such digital scalable interventions could be of particular usefulness in the treatment of PCS patients, given the large amount of patients affected worldwide [6].

Our study has strengths and limitations. Strengths are the sufficiently large and distinctly defined PCS patient sample, a healthy control group that did not differ from the PCS patients group in terms of sociodemographic (age, gender education, occupational level) and clinically relevant baseline variables (BMI, nicotine use, burden of comorbidities) and the application of extensive neuropsychological assessment using standardized, valid, and reliable test procedures including computerized testing. Our findings cannot be generalized to all PCS patients, as the sample was comprised of patients explicitly reporting cognitive deficits and seeking treatment. In addition, we cannot inform about potential spontaneous remission of cognitive dysfunction within the first months following COVID-19, as the first assessment took place on average around 1 year after initial infection.

In summary, the present study’s results align with the assumptions of the hypoarousal model of neurological PCS [12]. The deficit profile across established neuropsychological tests in 88 PCS patients indicates substantially reduced tonic alertness, and thus, general cognitive slowing, as a core and selective cognitive deficit affecting performance in time-critical tasks and measures. This first longitudinal study assessing neurocognitive deficits in PCS patients with particular focus on processing speed revealed that the tonic alertness dysfunction impairment persists without any signs of amelioration over a 6-month period. The impairment was measurable using a computerized simple-response task, which—in the version applied here or with similar alternatives—might turn out to be an economic and appropriate neuropsychological tool for empirical measurement of neurocognitive dysfunction in PCS syndrome.

### Supplementary Information

Below is the link to the electronic supplementary material.

Supplementary file1 (DOCX 364 KB)

## Data Availability

Upon a reasonable request, the corresponding author can provide the data supporting the findings of this study.
