# Supplementary material for: Persistent cognitive slowing in post-COVID patients: longitudinal study over 6 months
Source: J Neurol. 2023 Nov 7;271(1):46–58. doi: 10.1007/s00415-023-12069-3 (PMC10769987; doi:10.1007/s00415-023-12069-3)
Supplement: Supplementary file 1 — Supplementary file1 (DOCX 364 KB) [file 415_2023_12069_MOESM1_ESM.docx]

**Persistent cognitive slowing in post-COVID patients? Longitudinal study over six months**

Eva Maria Martin^1^, Annie Srowig^1^, Isabelle Utech^1^, Simon Schrenk^1^, Fabian Kattlun^1^, Monique Radscheidt^1^, Stefan Brodoehl^1^, Peter Bublak^1^, Matthias Schwab^1^, Christian Geis^1^, Bianca Besteher^2,3^, Philipp A. Reuken^4,^ Andreas Stallmach^4,5^, & Kathrin Finke^1,5,6^

^1^Department of Neurology, Jena University Hospital, Jena, Germany, ^2^Department of Psychiatry, Jena University Hospital, Jena, Germany, ^3^German Center for Mental Health (DZPG), ^4^Department of Internal Medicine IV (Gastroenterology, Hepatology and Infectious Diseases), Jena University Hospital, Jena, Germany, ^5^Center for Sepsis Control and Care, Jena University Hospital, Jena, Germany, ^6^Department of Psychology, Ludwig Maximilian University of Munich, Munich, Germany

**Supplementary Information**

1. **Comparison between non-hospitalized PCS patients during COVID-19 (*n* = 72) and healthy control participants (*n* = 50) at baseline**

**Table 1** Comparison of age-normed scores of non-hospitalized PCS patients and healthy control participants in the different test battery tasks and domains at baseline

| **Cognitive measures** | **Non-hospitalized PCS patients (*n* = 72)** | **Healthy control participants (*n* = 50)** | **Comparison between groups** | |
| --- | --- | --- | --- | --- |
| **TAP** - T-scores (*M* = 50, *SD* = 10) | *Mdn* (*IQR*)  Percentage below > 1.5 *SD*  from the norm’s mean | *Mdn* (*IQR*)  Percentage below  > 1.5 *SD*  from the norm’s mean | *p* | *r* |
| **RT Tonic Alertness** | 33.00 (8.00) | 43.50 (11.00) | <.001 | .50 |
| **RT Phasic Alertness** | 33.00 (11.00) | 42.00 (10.00) | <.001 | .51 |
| **RT Divided Attention (Auditory)** | 34.00 (8.00) | 45.60 (15.00) | <.001 | .42 |
| **RT Divided Attention (Visual)** | 43.00 (15.00) | 51.50 (12.00) | .001 | .31 |
| **RT Inhibition** | 41.00 (17.00) | 49.00 (12.00) | .001 | .30 |
| **Errors Divided Attention** | 55.00 (16.00) | 49.00 (18.00) | .096 |  |
| **Errors Inhibition** | 53.00 (1.00) | 53.00 (1.00) | .026 | .21 |

| **S-NAB** – standard scores (*M* = 100, *SD* = 15) | *M* (*SD*)  Percentage below  > 1.5 *SD*  from the norm’s mean | *M* (*SD*)  Percentage below  > 1.5 *SD*  from the norm’s mean | *p* | *d* |
| --- | --- | --- | --- | --- |
| **Attention** | 82.53 (14.98) | 99.16 (14.10) | <.001 | 1.14 |
| **Language** | 102.04 (17.74) | 107.06 (11.31) | .064 |  |
| **Memory** | 104.57 (14.73) | 110.66 (12.63) | .021 | 0.44 |
| **Perception** | 94.25 (14.12) | 103.32 (12.57) | <.001 | 0.67 |
| **Executive Functions** | 88.53 (16.25) | 101.70 (12.79) | <.001 | 0.88 |

*Note*. *Mdn* and *IQR* are reported for Wilcoxon rank-sum tests, *M* and *SD* for t-tests. FDR-adjusted *p*-values. Effect sizes (Cohen's *d* for t-tests, *r* for Wilcoxon rank-sum tests) were only reported for significant tests with *d* ≥ 0.2; *r* ≥ 0.1 indicating small, *d* ≥ 0.5; *r* ≥ 0.3 indicating moderate, and *d* ≥ 0.8; *r* ≥ 0.5 indicating large effects. TAP = Test for Attentional Performance; S-NAB = Neuropsychological Assessment Battery Screening Module.

1. **Regression analyses**

**Table 2** Multiple regression analyses with the group variable, baseline sociodemographics and clinical characteristics as predictors for test performance at baseline across PCS patients and healthy control participants (n = 138)

| **Multiple regression results** | | | | | |
| --- | --- | --- | --- | --- | --- |
| **Predictor** | **b** | **b** | ***p*** | **FDR-adj. *p*** | **Fit** |
| **Model 1: RT Tonic alertness at baseline as criterion** | | | | | |
| **(Intercept)** | 52.57 |  | <.001 | **<.001** |  |
| **Group** | -8.54 | -0.42 | <.001 | **<.001** |  |
| Age | -0.10 | -0.12 | .208 | .468 |  |
| Sex | -0.33 | -0.01 | .955 | .955 |  |
| Education | -0.25 | -0.03 | .793 | .892 |  |
| Occupation | 1.58 | 0.08 | .388 | .582 |  |
| Nicotine use | -4.62 | -0.18 | .039 | .117 |  |
| CCI | 0.67 | 0.06 | .503 | .646 |  |
| BMI | -0.17 | -0.09 | .294 | .529 |  |
|  |  |  |  |  | *R*^2^ = .220***  *R*^2^adj.=.17  95% CI[.13,.44] |

| **Model 2: RT Phasic alertness at baseline as criterion** | | | | | |
| --- | --- | --- | --- | --- | --- |
| **(Intercept)** | 51.07 |  | <.001 | **<.001** |  |
| **Group** | -7.49 | -0.45 | <.001 | **<.001** |  |
| Age | -0.04 | -0.06 | .545 | .796 |  |
| Sex | 0.98 | 0.05 | .456 | .795 |  |
| Education | 0.25 | 0.03 | .708 | .837 |  |
| Occupation | -0.43 | -0.02 | .837 | .171 |  |
| Nicotine use | -3.20 | -0.15 | .076 | .797 |  |
| CCI | -0.29 | -0.03 | .643 | .646 |  |
| BMI | -0.29 | -0.18 | .031 | .093 |  |
|  |  |  |  |  | *R*^2^ =.250***  *R*^2^adj.=.543  95% CI[.18,.43] |
| **Model 3: RT Divided Attention Auditory at baseline as criterion** | | | | | |
| **(Intercept)** | 45.73 |  | <.001 | **<.001** |  |
| **Group** | -8.35 | -0.40 | <.001 | **<.001** |  |
| Age | -0.10 | -0.11 | .263 | .473 |  |
| Sex | 1.10 | 0.04 | .503 | .503 |  |
| Education | -0.66 | -0.07 | .495 | .503 |  |
| Occupation | 1.54 | 0.07 | .414 | .503 |  |
| Nicotine use | -3.06 | -0.11 | .189 | .472 |  |
| CCI | 0.96 | 0.09 | .359 | .503 |  |
| BMI | 0.22 | 0.11 | .210 | .473 |  |
|  |  |  |  |  | *R*^2^ =.183***  *R*^2^adj.=.13  95% CI[.11,.38] |

| **Model 4: RT Divided Attention Visual at baseline as criterion** | | | | | |
| --- | --- | --- | --- | --- | --- |
| **(Intercept)** | 42.40 | -0.24 | .003 | **.027** |  |
| **Group** | -5.30 | -0.03 | .008 | **.036** |  |
| Age | -0.03 | -0.02 | .757 | .903 |  |
| Sex | -0.61 | 0.08 | .850 | .903 |  |
| Education | 0.76 | 0.01 | .456 | .903 |  |
| Occupation | 0.22 | -0.06 | .895 | .903 |  |
| Nicotine use | -1.62 | 0.04 | .535 | .903 |  |
| CCI | 0.42 | 0.01 | .734 | .903 |  |
| BMI | 0.03 | -0.24 | .903 | .903 |  |
|  |  |  |  |  | *R*^2^ =.069  *R*^2^adj.=.01  95% CI[.00,.24] |
| **Model 5: RT Inhibition at baseline as criterion** | | | | | |
| **(Intercept)** | 35.78 |  | .008 | **.036** |  |
| **Group** | -7.11 | -0.33 | <.001 | **<.001** |  |
| Age | -0.00 | -0.00 | .974 | .974 |  |
| Sex | 3.17 | 0.12 | .133 | .399 |  |
| Education | 0.79 | 0.08 | .411 | .740 |  |
| Occupation | 0.68 | 0.03 | .728 | .936 |  |
| Nicotine use | -2.61 | -0.09 | .293 | .659 |  |
| CCI | -0.17 | -0.01 | .838 | .942 |  |
| BMI | 0.10 | 0.05 | .611 | .917 |  |
|  |  |  |  |  | *R*^2^ =.135*  *R*^2^adj.=.087  95% CI[.09,.32] |

| **Model 6: Errors Divided Attention at baseline as criterion** | | | | | |
| --- | --- | --- | --- | --- | --- |
| **(Intercept)** | 52.64 |  | .000 | **<.001** |  |
| Group | 1.44 | 0.07 | .419 | .623 |  |
| Age | -0.05 | -0.06 | .541 | .623 |  |
| Sex | 2.97 | 0.12 | .204 | .459 |  |
| Education | -0.54 | -0.06 | .578 | .623 |  |
| Occupation | 1.14 | 0.05 | .623 | .623 |  |
| Nicotine use | -4.29 | -0.16 | .089 | .267 |  |
| CCI | -2.24 | -0.21 | .032 | .144 |  |
| BMI | 0.11 | 0.06 | .537 | .623 |  |
|  |  |  |  |  | *R*^2^ =.089  *R*^2^adj.=.026  95% CI[.00,.27] |
| **Model 7: Errors Inhibition at baseline as criterion** | | | | | |
| **(Intercept)** | 50.94 |  | <.001 | **<.001** |  |
| **Group** | -2.81 | -0.27 | .002 | **.009** |  |
| Age | -0.07 | -0.15 | .110 | .165 |  |
| Sex | -0.40 | -0.03 | .729 | .806 |  |
| Education | -0.12 | -0.03 | .806 | .806 |  |
| Occupation | 1.83 | 0.17 | .076 | .162 |  |
| Nicotine use | 2.03 | 0.15 | .090 | .162 |  |
| CCI | 1.03 | 0.19 | .038 | .114 |  |
| BMI | 0.05 | 0.05 | .597 | .768 |  |
|  |  |  |  |  | *R*^2^ =.163*  *R*^2^adj.=.026  95% CI[.11,.33] |

| **Model 8: Attention at baseline as criterion** | | | | | |
| --- | --- | --- | --- | --- | --- |
| (Intercept) | 41.71 |  | .021 | .063 |  |
| **Group** | -17.29 | -0.51 | <.001 | **<.001** |  |
| Age | 0.08 | 0.05 | .516 | .516 |  |
| Sex | -1.97 | -0.05 | .512 | .516 |  |
| Education | 2.02 | 0.13 | .129 | .232 |  |
| **Occupation** | 8.05 | 0.23 | .007 | **.031** |  |
| Nicotine use | 2.37 | 0.05 | .483 | .516 |  |
| CCI | 1.43 | 0.08 | .302 | .453 |  |
| BMI | 0.51 | 0.15 | .045 | .101 |  |
|  |  |  |  |  | *R*^2^ =.364**  *R*^2^adj.=.326  95% CI[.28,.51] |
| **Model 9: Language at baseline as criterion** | | | | | |
| **(Intercept)** | 96.54 |  | <.001 | **<.001** |  |
| Group | -4.64 | -0.14 | .100 | .300 |  |
| Age | -0.07 | -0.05 | .700 | .858 |  |
| Sex | -4.16 | -0.11 | .315 | .709 |  |
| Education | 0.32 | 0.02 | .812 | .858 |  |
| Occupation | 5.49 | 0.16 | .100 | .300 |  |
| Nicotine use | -0.98 | -0.02 | .790 | .858 |  |
| CCI | 0.45 | 0.03 | .858 | .858 |  |
| BMI | -0.10 | -0.03 | .733 | .858 |  |
|  |  |  |  |  | *R*^2^ =.068  *R*^2^adj.=.009  95% CI[.00,.27] |

| **Model 10: Memory at baseline as criterion** | | | | | |
| --- | --- | --- | --- | --- | --- |
| **(Intercept)** | 68.09 |  | <.001 | **<.001** |  |
| **Group** | -6.75 | -0.23 | .009 | **.041** |  |
| Age | 0.15 | 0.12 | .233 | .412 |  |
| Sex | -3.36 | -0.10 | .209 | .412 |  |
| Education | 1.49 | 0.11 | .275 | .412 |  |
| Occupation | 7.13 | 0.24 | .021 | .063 |  |
| Nicotine use | 1.34 | 0.04 | .678 | .762 |  |
| CCI | -0.46 | -0.03 | .794 | .794 |  |
| BMI | 0.17 | 0.06 | .487 | .626 |  |
|  |  |  |  |  | *R*^2^ =.152*  *R*^2^adj.=.009  95% CI[.10,.31] |
| **Model 11: Perception at baseline as criterion** | | | | | |
| **(Intercept)** | 99.16 |  | <.001 | **<.001** |  |
| **Group** | -8.41 | -0.29 | .001 | **.005** |  |
| Age | 0.00 | 0.00 | .967 | .967 |  |
| Sex | 2.49 | 0.07 | .382 | .573 |  |
| Education | -1.48 | -0.11 | .264 | .573 |  |
| Occupation | 6.61 | 0.22 | .025 | .075 |  |
| Nicotine use | 3.11 | 0.08 | .356 | .573 |  |
| CCI | -0.82 | -0.05 | .540 | .610 |  |
| BMI | 0.15 | 0.05 | .542 | .610 |  |
|  |  |  |  |  | *R*^2^ =.127*  *R*^2^adj.=.074  95% CI[.08,.31] |

| **Model 11: Executive functions at baseline as criterion** | | | | | |
| --- | --- | --- | --- | --- | --- |
| **(Intercept)** | 50.32 |  | .005 | **.023** |  |
| **Group** | -14.12 | -0.43 | <.001 | **<.001** |  |
| Age | 0.08 | 0.06 | .586 | .586 |  |
| Sex | -2.82 | -0.07 | .241 | .310 |  |
| Education | 2.48 | 0.17 | .078 | .144 |  |
| Occupation | 5.75 | 0.17 | .080 | .144 |  |
| Nicotine use | 4.86 | 0.11 | .152 | .228 |  |
| CCI | 2.50 | 0.15 | .058 | .144 |  |
| BMI | 0.25 | 0.08 | .331 | .372 |  |
|  |  |  |  |  | *R*^2^ =.297*  *R*^2^adj.=.238  95% CI[.23,.47] |

*Note.* *n* = 135 PCS patients and healthy control participants. b = unstandardized regression weights, beta = standardized regression weights. Square brackets enclose bootstrapped (1,000 samples) confidence intervals (LL = lower limit, UL = upper limit). FDR-adjusted *p*-values. RT = reaction time. Tonic alertness, Phasic alertness, Divided attention and Inhibition in T-Scores (*M* = 50, *SD* = 10). Attention, Language, Memory, Perception and Executive functions in standard scores (*M* = 100, *SD* = 15). Group = PCS patients vs. healthy control participants. Education in school years. Occupation = Academic vs. Apprenticeship vs. no apprenticeship needed. CCI = Charlson Comorbidity Index. BMI = Body mass index.

1. **Correlational analyses**
   1. *Association between neurocognitive performance, self-rated fatigue, depressive symptoms, time from COVID-19, hospitalization, sex and age at baseline*

**Table 3** Correlation between neurocognitive performance, self-rated fatigue, depressive symptoms, time from COVID-19, hospitalization, sex and age at baseline within the PCS patient group (*n* = 88) at baseline

|  | **Fatigue** | **Depressive symptoms** | **Hospitali-zation** | **Time from COVID-19** | **BMI** | **Nicotine use** | **CCI** | **Age** | **Sex** | **Education** | **Occupation** |
| --- | --- | --- | --- | --- | --- | --- | --- | --- | --- | --- | --- |
| **TAP** (T-scores, *M* = 50, *SD* = 10) | | | | | | |  |  |  |  |  |
| **RT Tonic Alertness** | **-.408^**^** | -.280 | -.021 | -.090 | -.214 | -.069 | -.032 | -.110 | -.092 | .122 | .153 |
| **RT Phasic Alertness** | **-.376^*^** | -.244 | -.015 | -.156 | -.310 | -.099 | -.011 | -.119 | -.031 | .137 | .110 |
| **RT Divided Attention (Auditory)** | **-.414^**^** | -.231 | .104 | .089 | -.048 | -.111 | .116 | .034 | .067 | .036 | .020 |
| **RT Divided Attention (Visual)** | **-.459^**^** | -.222 | .161 | -.003 | -.074 | -.037 | .065 | .035 | .058 | .119 | .043 |
| **RT Inhibition** | -.315 | -.183 | .005 | .074 | .038 | -.124 | -.067 | -.032 | .069 | .055 | .070 |
| **Errors Divided Attention** | -.023 | .053 | -.332 | -.030 | .061 | -.202 | -.307 | -.135 | .037 | .169 | .084 |
| **Errors Inhibition** | .163 | .072 | -.007 | .023 | -.016 | .177 | .051 | -.084 | .029 | .081 | .164 |
| **S-NAB** (standard scores, *M* = 100, *SD* = 15) | | | | | | |  |  |  |  |  |
| **Attention** | -.134 | -.222 | -.036 | .024 | .094 | -.052 | .042 | .113 | -.052 | .290 | .279 |
| **Language** | -.227 | -.148 | -.093 | .039 | -.083 | -.065 | -.126 | -.084 | -.029 | .139 | .169 |
| **Memory** | -.019 | -.029 | -.106 | -.040 | .016 | -.039 | .007 | .095 | -.164 | .069 | .279 |
| **Perception** | -.132 | -.127 | .070 | -.188 | .022 | .085 | .018 | -.054 | .060 | .100 | .100 |
| **Executive functions** | .018 | -.186 | -.089 | .019 | -.036 | -.004 | .108 | -.063 | -.046 | .262 | .239 |

*Note:* Pearson/point-biserial correlation coefficients. RT = reaction times. Fatigue = Fatigue Assessment Scale (FAS) score, depressive symptoms = Hospital Anxiety and Depression Scale (HADS-D) depression subscore, hospitalization (during COVID-19) operationalized as 0 = no hospitalization, 1 = normal ward, 2 = intensive care unit, time from COVID-19 in days; age in years. FDR-adjusted *p*.* *p* < .05; ** *p* < .01


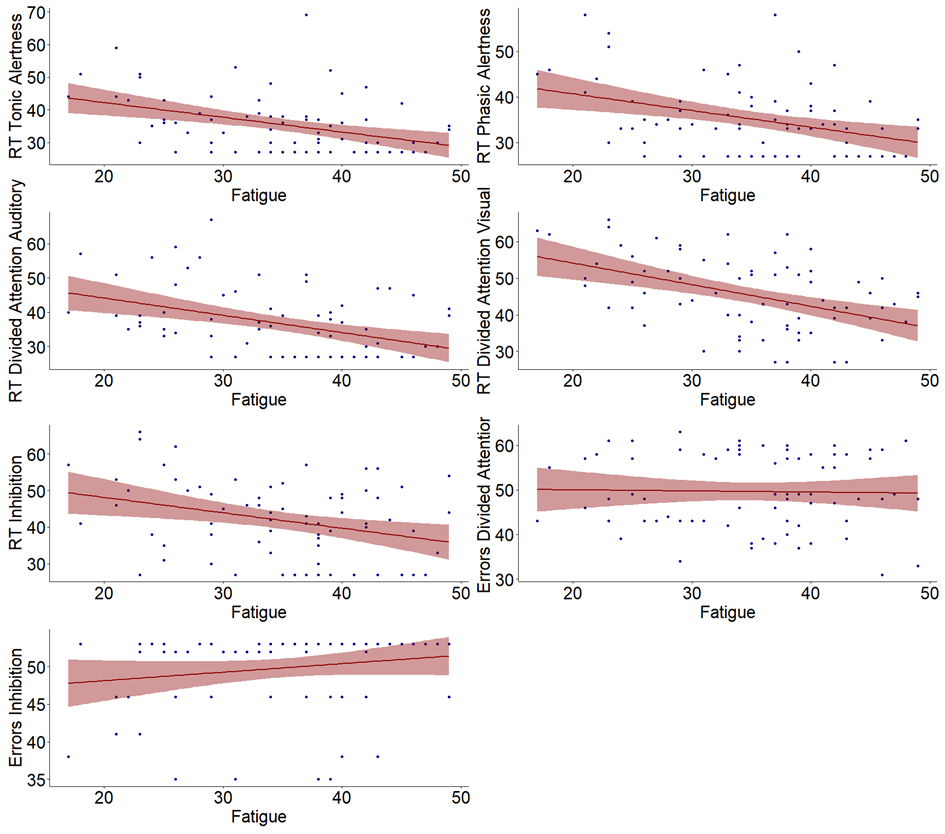


**Fig. 1** Scatterplots depicting self-rated fatigue and neurocognitive performance in TAP-battery tasks at baseline.


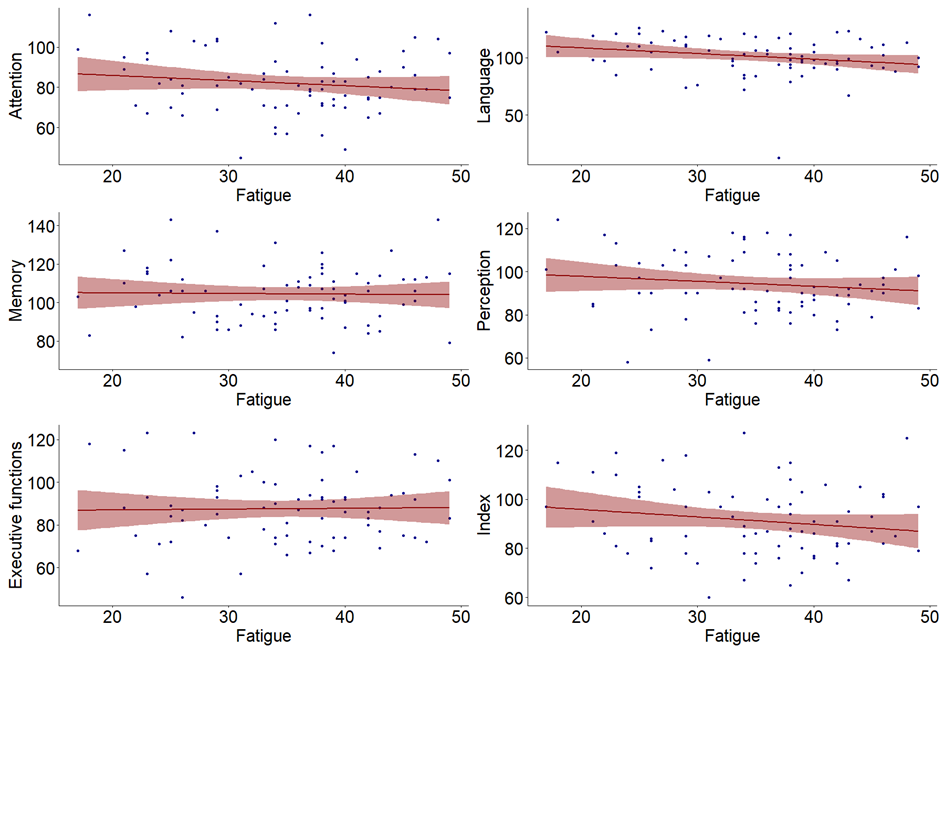


**Fig. 2** Scatterplots depicting self-rated fatigue and neurocognitive performance in S-NAB-battery domains at baseline.

- 1. *Correlation between neurocognitive performance at baseline and neurocognitive performance at six-month follow-up*

**Table 4** Correlation between neurocognitive performance at baseline and neurocognitive performance at six-month follow-up

|  |  | **Baseline** | | | | | | | | | | | | |
| --- | --- | --- | --- | --- | --- | --- | --- | --- | --- | --- | --- | --- | --- | --- |
| **Six-month follow-up** |  | **1.** | **2.** | **3.** | **4.** | **5.** | **6.** | **7.** | **8.** | **9.** | **10.** | **11.** | **12.** | **13.** |
|  | **TAP (T-scores)** |  |  |  |  |  |  |  |  |  |  |  |  |  |
|  | **1. RT Tonic Alertness** | .69** | .68** | .28** | .39** | .33** | .03 | 003 | .35** | .19 | .14 | .11 | .30** | .31** |
|  | **2. RT Phasic Alertness** | .56** | .60** | .19 | .40** | .32** | .04 | -0.01 | .33** | .18 | .07 | .12 | .29** | .27* |
|  | **3. RT Divided Attention (Auditory)** | .34** | .32** | .54** | .42** | .29** | -.08 | .24* | .36** | .22* | .04 | .07 | .27* | .29** |
|  | **4. RT Divided Attention (Visual)** | .29** | .33** | .38** | .55** | .26* | -.09 | .26* | .34** | .27* | .17 | .10 | .16 | .30** |
|  | **5. RT Inhibition** | .268* | .26* | .37** | .38** | .49** | -.18 | .15 | .11 | .17 | .05 | .06 | .09 | .11 |
|  | **6. Errors Divided Attention** | .05 | -.02 | -.05 | -0.02 | -.05 | .35** | .27** | .32** | .06 | .14 | 0.11 | .21 | .27* |
|  | **7. Errors Inhibition** | .20 | .14 | .09 | .30** | .02 | -.01 | .26* | .24* | .25* | .15 | 0.10 | .23* | .32** |
|  | **NAB (standard scores)** |  |  |  |  |  |  |  |  |  |  |  |  |  |
|  | **8. Attention** | .37** | .29** | .19 | .36** | .09 | .03 | .29** | .64** | .34** | .21* | .22* | .54** | .57** |
|  | **9. Language** | .25* | .22* | .16 | .35** | .11 | -.19 | .24* | .29** | .29** | .11 | .14 | .23* | .33** |
|  | **10. Memory** | .12 | .06 | -.20 | 0.15 | -.05 | .04 | .15 | .26* | .32** | .57** | .28** | .27* | .56** |
|  | **11. Perception** | -.02 | .05 | -.04 | 0.08 | -.06 | -.04 | .13 | .07 | .16 | .29** | .25* | .15 | .30** |
|  | **12. Executive functions** | .24* | .23* | .15 | .31** | .01 | .09 | .34** | .48** | .35** | .16 | .32** | .72** | .62** |

*Note*. FDR-adjusted *p*-values. ***p* < .01; **p* < .05. RT = reaction times. RTs and errors expressed in T-scores (*M* = 50, *SD* = 10). Attention, language, memory, perception and executive functions expressed in standard scores (*M* = 100, *SD* = 15).

1. **Results based on non-imputed data**

### *Comparison of neurocognitive performance, self-rated fatigue and self-rated depressive symptoms between assessment at baseline and at six-month follow-up based on non-imputed data*

Performance of PCS patients between assessment at baseline and six-month follow-up only improved in the domains of perception and executive functions with small effect sizes, while there was no difference in performance between baseline and six-month follow-up for tonic alertness nor further cognitive measures. Also, neither self-rated fatigue nor depressive symptoms differed between baseline and six-month follow-up in the PCS patient group (see Table 3).

**Table 3**: Comparison of PCS patient’s performance in the tonic alertness task and further cognitive tasks at baseline and at six-month follow-up (non-imputed data)

| **Cognitive measures** | **Baseline** | **six-month**  **follow-up** | ***p*** | ***Effect size*** |
| --- | --- | --- | --- | --- |
| **TAP (T-scores)** | *Mdn (IQR)* | *Mdn (IQR)* |  |  |
| RT Tonic Alertness | 34.00 (8.00) | 33.00 (11.00) | .234 |  |
| RT Phasic Alertness | 33.00 (12.00) | 33.00 (11.00) | .173 |  |
| RT Divided Attention (Auditory) | 35.00 (13.00) | 34.00 (17.00) | .693 |  |
| RT Divided Attention (Visual) | 44.00 (15.00) | 46.00 (18.00) | .266 |  |
| RT Inhibition | 4150 (17.00) | 39.00 (18.00) | .752 |  |
| Errors Divided Attention | 49.00 (15.00) | 55.00 (16.00) | .497 |  |
| Errors Inhibition | 53.00 (6.00) | 53.00 (1.00) | .357 |  |
| **S-NAB (standard scores)** | *M (SD)* | *M (SD)* |  |  |
| Attention | 82.52 (14.41) | 80.29 (18.12) | .408 |  |
| Language | 101.65 (17.34) | 103.36 (10.93) | .484 |  |
| Memory | 104.09 (14.34) | 103.36 (16.64) | .752 |  |
| **Perception** | **94.72 (13.77)** | **101.62 (12.93)** | **013** | ***d* = .31** |
| **Executive functions** | **87.61 (15.50)** | **91.81 (16.07)** | **.032** | ***d* = .40** |
| **Clinical variables** |  |  |  |  |
| Fatigue | 37.00 (11.00) | 35.00 (16.00) | .473 |  |
| Depressive symptoms | 7.00 (5.00) | 7.00 (7.00) | .102 |  |

*Note*. *n*  = 77 PCS patients. *Mdn* and *IQR* are reported for Wilcoxon signed-rank tests, *M* and *SD* for t-tests. FDR-adjusted *p*-values. Effect sizes (Cohen's *d* for t-tests, *r* for Wilcoxon rank-sum tests) were only reported for significant tests with *d* ≥ 0.2; *r* ≥ 0.1 indicating small, *d* ≥ 0.5; *r* ≥ 0.3 indicating moderate, and *d* ≥ 0.8; *r* ≥ 0.5 indicating large effects. Results for significant tests in bold. Fatigue = Fatigue Assessment Scale (FAS) score, Depressive symptoms = Hospital Anxiety and Depression Scale (HADS-D) depression subscore.
